# Development and characterization of thirteen novel microsatellite markers for use in Greenland sharks (*Somniosus microcephalus*), with cross-amplification in Pacific sleeper sharks (*Somniosus pacificus*)

**DOI:** 10.1186/s13104-021-05447-5

**Published:** 2021-01-19

**Authors:** Meaghan A. Swintek, Ryan P. Walter

**Affiliations:** California State University, Fullerton, USA

**Keywords:** Somniosus, Greenland shark, Population genetics, Microsatellites, Sleeper shark

## Abstract

**Objective:**

The objectives of this work are to isolate, develop, and characterize polymorphic microsatellite markers for use in Greenland sharks (*Somniosus microcephalus*).

**Results:**

Thirteen microsatellite loci were successfully amplified and yielded multi-locus genotypes for 36 *S. microcephalus* individuals from Grise Fjord (n = 16) and Svalbard (n = 20). Each locus yielded between 2 and 9 alleles and observed heterozygosity ranged from 0.11 to 0.70 when estimated across both sites. One locus and three loci deviated from HWE following Bonferroni correction, for individuals sampled from Grise Fjord and Svalbard, respectively. Cross-amplification was successful at every locus for five of the ten *S. pacificus* individuals.

## Introduction

Greenland sharks (*Somniosus microcephalus*) are long lived [1] and presumably late to mature [2] sharks capable of extensive migration [3] in the North Atlantic to Arctic marine environments. Previous molecular genetic work successfully differentiated Greenland sharks from other species in *Somniosus* [4] and revealed hybridization with Pacific sleeper sharks (*Somniosus pacificus*) [5, 6], however, knowledge of their population-level genetic variation is yet to be described.

Highly variable molecular genetic markers, such as microsatellites, can provide data to characterize population genetic structure, and have proven useful for describing spatial genetic variation in widely distributed elasmobranchs [7–9]. Here, we isolate, develop, and describe polymorphic microsatellite loci to provide molecular genetic markers for assessing the population-level genetic variation in the Greenland shark. Thirteen markers were identified as possible candidate loci for population analyses in Greenland shark samples collected from two locations in the Arctic. We also explored marker cross-amplification in Pacific sleeper sharks, providing evidence of utility in other *Somniosus* species.

## Main text

### Methods

Primer sets for 50 candidate microsatellite loci were constructed from reduced-representation genomic DNA sequence data from a single *Somniosus microcephalus* individual sampled from Resolute Bay (SRA accession: PRJNA655731). Genomic DNA from this individual was prepared using the QIAgen DNeasy Tissue Kit (Valencia, CA, USA) and shipped to Global Biologics (Columbia, MO) where a library was created with SPRI selection targeting 450 bp inserts. The library was pooled with other species libraries and sequenced on an Illumina HiSeq 2500. Approximately 4 million 250 bp PE reads were recovered; from these, tetrasat motifs with a minimum of five uninterrupted repeating motifs (e.g. GACAGACAGACAGACA) were targeted using STR finder in Galaxy [10] and PCR primer regions flanking each motif were identified using PRIMER3 [11]. A total of 36 *S. microcephalus* sampled from two sites (Grise Fjord: n = 16; Svalbard: n = 20) were used to characterize and optimize the prospective microsatellite markers, and an additional ten *S. pacificus* individuals were used to test for cross-species amplification. For all individuals, genomic DNA was extracted and purified from fin tissue stored in either ethanol or RNA*later* (Invitrogen, Carlsbad, CA, USA), using the Promega Wizard Genomic DNA Extraction Kit (Promega Corp, Madison, WI, USA), according to the manufacturer’s instructions.

PCR reactions occurred in 12.5 µL volumes containing 1.25 µL 10X PCR reaction buffer with 15 mM magnesium chloride (GenScript, Piscataway, NJ, USA), 0.25 µL 40 mM dNTP’s (APEX BioResearch Products), 0.25 µL 10 µM forward primer, 0.25 µL 10 µM reverse primer, 5 U Taq Polymerase (GenScript, Piscataway, NJ, USA), and 0.5 µL genomic DNA, with one locus (Smic2) requiring an additional 0.625 µL 15 mM magnesium chloride per reaction. Thermal-cycler conditions consisted of an initial denaturation at 94 °C for 2 min, followed by 30 cycles of 94 °C for 30 s (denaturation), 52–57 °C for 30 s (annealing) depending on marker identity (Table 1), 72 °C for 1 min (extension), and a final step at 72 °C for 1 min and 30 s. Three loci (Smic2, Smic24, Smic31) required half the time within each cycle (i.e., reduced from 30 to 15 s and 1 min to 30 s). PCR products were visualized on a 1.5% agarose gels for estimated size confirmation, then measured for precise fragment lengths using a Fragment Analyzer (Advanced Analytic Technologies, Inc., Ankeny, IA, USA) according to the manufacturer’s protocol. Fragment lengths were scored using PROsize 3.0 software (Advanced Analytic Technologies, Inc., Ankeny, IA, USA), and were binned across samples to account for variation between plate runs. All loci were analyzed for null alleles, stuttering, or large allele dropout using Micro-Checker 2.2.3 [12]. The total number of alleles and observed and expected heterozygosity were calculated in GenAlEx 6.512b [13]. Deviations from Hardy–Weinberg equilibrium (HWE) and detection of linkage disequilibrium among all loci pairs was determined in Genepop 4.2 [14] for each site separately.

**Table 1:** Thirteen novel microsatellite markers developed for use in *Somniosus microcephalus* (n = 35)

Locus	Sequence (5′—> 3′)	T_A_ (°C)	Range (bp)	Motif	N_A_
Smic1	F: TGCCTAGTAGACGCCCCTAA	52	157–189	CAGA	7
	R: TGTTCCCAGATGTGTGCATT				
Smic2	F: GCCTAAGCCACCCTCCTAAT	57	159–167	ACAG	2
	R: CTCCGGCATCTCCACACTAT				
Smic4	F: TATTTAGTCCCAGCAGTGCG	55	205–233	TGAC	6
	R: ACTTCGGCGACCATGTTCTA				
Smic5^a^	F: TGTTTCAGGAATAGGGATGCC	55	224–244	TCAG	4
	R: CAATCATTTATCTTGTGGAGCCA				
Smic10	F: ATGCCTATGACACTCCCCTG	52	176–204	GACA	6
	R: ACCTGCCACCCGATTAGTAA				
Smic12	F: TGTCCGACCGAAACGTAAAT	52	173–189	AGAC	3
	R: CCCTCAGCAGAACCATTCAT				
Smic13	F: CCCATAAACAGCGAATGACC	55	152–164	AGAC	3
	R: GCCTTTGAACCAAGGACAGA				
Smic15	F: ATGCTTAGGACGGTTCTGGA	52	196–220	AGAC	6
	R: ATCCCTCATCCTGTGGACTG				
Smic16	F: CAGTGACAAACATCCCCAAA	52	220–236	ATAG	5
	R: AAACAGCCTTTCCCCGTCTA				
Smic18	F: ACGTAAATACGCCGATGACC	52	212–224	CAGA	4
	R: GGCCATGAACTTATCCTCCA				
Smic20	F: TCCGAACTCTTTTGGCTGAC	55	243–259	GACA	4
	R: CGTTCTCAGCTCAGGGATCT				
Smic24	F: TCACTGGTCCGTAATCGTCA	55	205–213	GACA	4
	R: CCACATCTTCCGGCTCTAAA				
Smic31	F: ATACGCTTATGACCGCTCCG	57	243–259	GACA	3
	R: GTCCAAAACACAGAGCAGGG				

Includes: Locus name, forward (F) and reverse (R) primer sequences, annealing temperature (T_A_), fragment length range in base pairs, repeat tetrasat motif, total number of alleles (N_A_)

^a^ indicates presence of null alleles

### Results and discussion

Of the microsatellite loci screened in *Somniosus microcephalus* samples, 24 successfully amplified consistently and yielded indications of allelic polymorphism on agarose gels. Following initial agarose screening, 13 loci were further identified through fragment analysis as having clear fragment length peaks, and were then scored among 36 individuals. All other loci were deemed not suitable due to either monomorphic allele scoring, weak amplification, or the production of multiple (> 2) fragments following PCR and thermal-cycler optimizations (Additional file 1: Table S1).

Each locus produced two to seven alleles within each site, with observed and expected heterozygosity ranging from 0.063 to 0.750 and 0.117 to 0.770, respectively (Table 2). Four loci failed to conform to HWE following Bonferroni correction for multiple tests: Smic 24 at GF, and Smic1, Smic4, Smic16 at SV. Of the 13 loci, a single locus (Smic5) displayed signs of stuttering and possible scoring error, and homozygote excess in both sampling sites. Two additional loci (Smic4, Smic24) displayed homozygote excess in Grise Fjord samples as indicated by Microchecker.

**Table 2:** Mean descriptive statistics within each site at the developed microsatellite loci

	Grise Fjord (GF)				Svalbard (SV)		
*Locus*	N_A_	H_O_	H_E_		N_A_	H_O_	H_E_
Smic1	6	0.563	0.621		6	*0.500*	*0.666*
Smic2	2	0.125	0.117		2	0.150	0.219
Smic4	6	0.438	0.738		6	*0.500*	*0.614*
Smic5^a^	4	0.250	0.658		3	0.100	0.261
Smic10	5	0.500	0.523		6	0.600	0.736
Smic12	3	0.375	0.404		3	0.200	0.226
Smic13	3	0.375	0.314		3	0.300	0.261
Smic15	5	0.750	0.709		7	0.650	0.770
Smic16	5	0.688	0.645		4	*0.450*	*0.571*
Smic18	4	0.438	0.637		2	0.500	0.495
Smic20	3	0.188	0.361		2	0.200	0.420
Smic24	3	*0.063*	*0.354*		2	0.150	0.139
Smic31	3	0.438	0.361		3	0.500	0.406

Includes: number of alleles (N_A_), observed heterozygosity (H_O_) and expected (H_E_) heterozygosity for Grise Fjord (GF) and Svalbard (SV)

Italics values indicate significant departures from HWE following Bonferroni correction

^a^ indicates presence of null alleles

Exact tests performed for each site resulted in deviations from HWE for individuals at one locus from Grise Fjord and three loci from Svalbard (Table 2). Linkage disequilibrium was not present at any locus pair in either site following Bonferroni correction. Cross-amplification at each of the 13 loci was successful in 5 of the 10 *S. pacificus* samples using the same cycling conditions. Four samples failed to amplify at 1 locus (2 samples at Smic15; 1 sample at Smic18; 1 sample at Smic1) and one sample at 2 loci (Smic1 and Smic18).

Due to the low sample sizes, it is not possible to make statements regarding the biological significance versus sampling artifacts for departures from HWE, homozygosity excess, and presence of null alleles. Nonetheless, these markers will be useful for exploring genetic variation and stock structure in Greenland sharks throughout their distribution. Furthermore, cross-amplification in the closely related Pacific sleeper shark (*Somniosus pacificus*) extends potential utility to other sleeper shark species.

## Limitations

- Low sample size and/or a priori site-based population designation may be contributing to deviations from HWE, excess homozygosity, and presence of null alleles.

## Supplementary Information

**Additional file 1: Table S1.** Eleven additional microsatellite loci primer sets that were not further developed due to either lack of polymorphism or greater than two peaks during PCR amplification screening.

## Data Availability

The DNA sequences containing the microsatellite loci from which these primers were developed were submitted to Genbank (SRA accession: PRJNA655731). The datasets generated and/or analyzed during this study are available from the corresponding author upon request.

## Abbreviations

PCR: Polymerase chain reaction
HWE: Hardy–Weinberg Equilibrium
N_A_: Number of alleles
H_O_: Observed heterozygosity
H_E_: Expected heterozygosity
T_A_: Annealing temperature
bp: Base pairs
