# Supplementary material for: Development and characterization of thirteen novel microsatellite markers for use in Greenland sharks (Somniosus microcephalus), with cross-amplification in Pacific sleeper sharks (Somniosus pacificus)
Source: BMC Res Notes. 2021 Jan 19;14:28. doi: 10.1186/s13104-021-05447-5 (PMC7816355; doi:10.1186/s13104-021-05447-5)
Supplement: Supplementary file 1 — Additional file 1: Table S1. Eleven additional microsatellite loci primer sets that were not further developed due to either lack of polymorphism or greater than two peaks during PCR amplification screening. [file 13104_2021_5447_MOESM1_ESM.docx]

**Additional file 1: Table S1.**  Eleven additional microsatellite loci primer sets that were not further developed due to either lack of polymorphism or greater than two peaks during PCR amplification screening.

|  |  |  |  |  |  |
| --- | --- | --- | --- | --- | --- |
| **Locus** | **Primer Names** | | **Sequence (5-3)** |  | **Comments** |
| Smic6 | Smic_TCAC-5_62651_F | | AACTCCACCTGCTTCACTCA |  | Monomorphic |
|  | Smic_TCAC-5_62651_R | | TGCCGACCCTTCTCATTAGT |  |  |
| Smic7 | Smic_GCAT-5_57133_F | | GAGGGAAAACTCGCAGGTAA |  | Monomorphic |
|  | Smic_GCAT-5_57133_R | | TGGGATTGAATCGGCTTTGG |  |  |
| Smic8 | Smic_GATA-5_89584_F | | GGGAGACAGACAGATAGACAGA |  | Produced multiple peaks |
|  | Smic_GATA-5_89584_R | | ATCGTGCGACCAATTCAGG |  |  |
| Smic19 | Smic_GACA-5_95771_F | | CCGAACAATTTTTGTCTGACC |  | Produced multiple peaks |
|  | Smic_GACA-5_95771_R | | TCGCTCTCGTTCTCTCTTCC |  |  |
| Smic21 | Smic_GACA-5_93635_F | | GCCTATGACGCTCCCCTAAT |  | Monomorphic |
|  | Smic_GACA-5_93635_R | | GCCGCTGGGTTATGATGTA |  |  |
| Smic22 | Smic_GACA-5_46688_F | | AATGAGCGGAATTTTGAACG |  | Monomorphic |
|  | Smic_GACA-5_46688_R | | AAGGGTGTGCTGAAGAAGAGA |  |  |
| Smic23 | Smic_GACA-5_19807_F | | TGACTGTTCTGGAATCGTAAGC |  | Monomorphic |
|  | Smic_GACA-5_19807_R | | TGGAACATAGAAAGGGTGTGC |  |  |
| Smic32 | Smic_GACA-7_96191_F | | CTATGACGCTCCCCAATCAT |  | Monomorphic |
|  | Smic_GACA-7_96191_R | | GCGTTCATCATTAGAGCCTGT |  |  |
| Smic38 | Smic_TCTA-15_76860_F | | GGTCTGTCTGTGTCTGCCTT |  | Monomorphic |
|  | Smic_TCTA-15_76860_R | | AGCATTTGAACTGGGAACTGG |  |  |
| Smic41 | Smic_TCTA-19_22983_F | | TCCTCCCACCCACTCAAATAT |  | Produced multiple peaks |
|  | Smic_TCTA-19_22983_R | | ACCCGATCTCTGTTTACTTTACA |  |  |
| Smic50 | Smic_CAGA-9_57582_F | | TCGCAAGCTTTCTTTTGAGG |  | Produced multiple peaks |
|  | Smic_CAGA-9_57582_R | | CTCGTCGCTTTTCATGGATT |  |  |
|  |  |  |  |  |  |
